# Response to immune checkpoint inhibitor combination therapy in metastatic RET-mutated lung cancer from real-world retrospective data

**DOI:** 10.1186/s12885-024-11852-3

**Published:** 2024-02-05

**Authors:** Ningning Yan, Huixian Zhang, Shujing Shen, Sanxing Guo, Xingya Li

**Affiliations:** 1 Department of Medical Oncology, Zhengzhou University First Affiliated Hospital, 1st East Jianshe Road, Zhengzhou, Henan 450002 China; 2 Department of Radiation Oncology, Zhengzhou University First Affiliated Hospital, Zhengzhou, Henan 450002 China

**Keywords:** RET, Non-small cell lung cancer, PDL1, TMB

## Abstract

**Background:**

The impact of immune checkpoint inhibitors (ICIs) based treatments on non-small cell lung cancers (NSCLCs) with RET fusions remains poorly understood.

**Methods:**

We screened patients with RET fusions at the First Affiliated Hospital of Zhengzhou University and included those who were treated with ICIs based regimens for further analysis. We evaluated clinical indicators including objective response rate (ORR), disease control rate (DCR), progression-free survival (PFS), and overall survival (OS).

**Results:**

A total of 232 patients with RET fusions were included in the study. Of these, 129 patients had their programmed death-ligand 1 (PDL1) expression levels tested, with 22 patients (17.8%) having a PDL1 level greater than or equal to 50%. Additionally, tumor mutational burden (TMB) status was evaluated in 35 patients, with the majority (30/35, 85.8%) having a TMB of less than 10 mutations per megabase. Out of the 38 patients treated with ICI based regimens, the median PFS was 5 months (95% confidence interval [CI]: 2.4–7.6 months) and the median OS was 19 months (95% CI: 9.7–28.3 months) at the time of data analysis. Stratification based on treatment lines did not show any significant differences in OS (18 vs. 19 months, *p* = 0.63) and PFS (6 vs. 5 months, *p* = 0.86). The ORR for patients treated with ICIs was 26.3%. Furthermore, no significant differences were found for PFS (*p* = 0.27) and OS (*p* = 0.75) between patients with positive and negative PDL1 expression. Additionally, there was no significant difference in PD-L1 levels (*p* = 0.10) between patients who achieved objective response and those who did not.

**Conclusions:**

Patients with RET fusion positive NSCLCs may not benefit from ICI based regimens and therefore should not be treated with ICIs in clinical practice.

**Supplementary Information:**

The online version contains supplementary material available at 10.1186/s12885-024-11852-3.

## Introduction

Targeted therapy has revolutionized the treatment approach and extended the survival of advanced lung cancer with specific oncogenic drivers. In lung adenocarcinoma, around 50–60% of all lung cancer cases exhibit gene alterations with clinical significance that are associated with effective targeted treatments [1, 2]. As a result, molecular testing has become a standard part of the diagnostic process for advanced lung adenocarcinoma patients.

One emerging target in lung adenocarcinoma is the rearrangement of the proto-oncogene known as RET fusion, which accounts for only 1–2% of cases [3–5]. RET fusion leads to the constant activation of the RET tyrosine kinase, triggering the MAPK and PI3K oncogenic pathways [3–5]. Initially, the use of multikinase inhibitors (MTKis) like cabozantinib and vandetanib showed limited effectiveness in treating RET fusion-positive NSCLC [6–8]. However, the introduction of selective RET inhibitors (RETis) such as selpercatinib and pralsetinib has significantly improved the prognosis for advanced lung cancer patients with RET fusions [9–11].

Due to their better effectiveness and tolerability, the National Medical Products Administration (NMPA) and the Food and Drug Administration (FDA) have approved their use in RET fusion-positive NSCLC. However, the higher cost of these selective RET inhibitors restricts their availability to many Chinese patients.

Immune checkpoint inhibitors (ICIs) have significantly changed treatment approaches and improved the outlook for patients without identified driver mutations. However, their effectiveness tends to be lower for those with certain oncogenic driver mutations, such as EGFR or ALK, and these patients may experience more severe side effects [12–14]. Despite these challenges, the potential benefit of ICIs in individuals with other less common oncogenic drivers, like RET fusions, hasn’t been thoroughly investigated, largely because these patients aren’t usually excluded from clinical trials or specifically analyzed.

A handful of smaller retrospective studies have evaluated the use of ICIs in patients with non-small cell lung cancer (NSCLC) who have RET + mutations [12, 15, 16]. One study by Guisier et al. showed that these patients had a response to ICI monotherapy similar to those without driver mutations, with a 37.5% objective response rate (ORR) and a progression-free survival (PFS) of 7.6 months [16]. However, this study was limited to just nine patients with RET-rearranged NSCLC. Another study presented opposing findings, suggesting a poor response to ICI monotherapy in patients with RET fusions, evidenced by a 6.3% ORR and a PFS of only 2.1 months [12]. Baglivo et al. also indicated that RET rearrangements could predict a lack of response to ICI monotherapy in NSCLC, citing two cases that experienced rapid disease progression when treated with ICIs, even though they had high levels of programmed death-ligand 1 (PD-L1) [17]. Furthermore, Hedge’s group advised that, for RET positive tumors, non-ICI therapies were preferred over ICI-based treatments [18]. These studies taken together suggest that ICI monotherapy might not be the best approach for RET fusion positive NSCLC, but the potential of ICI-based combination treatments for these cancers still needs to be explored. A possible reason for this is that RET positive NSCLC is considered a “cold” tumor, similar to other types of oncogene-addicted NSCLCs, characterized by low PD-L1 expression and tumor mutational burden (TMB) [19].

Given the varied outcomes reported for ICI monotherapy in small case series of RET + NSCLC, the actual effectiveness of chemoimmunotherapy in RET fusion positive NSCLC is still unclear. This study aims to thoroughly characterize patients with RET fusion positive NSCLC in order to assess their clinical and biological features and to clarify the outcomes of ICI-based therapy in advanced NSCLC driven by RET addiction.

## Patients and methods

### Study design

This study is a single-center, retrospective analysis, involving real-world patients with RET fusion, identified from April 2017 to January 2022 at the First Affiliated Hospital of Zhengzhou University. The inclusion criteria were as follows: (1) patients aged 18 years or older; (2) all patients with RET fusion positive NSCLC; (3) patients who had undergone ICI-based therapy; (4) at least one measurable lesion as per the Response Evaluation Criteria in Solid Tumors, version 1.1 (RECIST v1.1); (5) OS of more than 3 months; (6) inclusion of patients who had received radiation or other therapies, or those who experienced recurrence post radical lung surgery. The main exclusion criteria included: (1) no previous ICI therapy; (2) tumors with mixed small cell lung cancer (SCLC) components; (3) acquired RET fusions due to targeting other oncogenes in prior treatments. The clinicopathological details and treatment data were gathered retrospectively from electronic health records (EHR).

### Data collection

The assessment of treatment responses was carried out in accordance with the Response Evaluation Criteria in Solid Tumors, version 1.1 (RECIST 1.1). Data on clinicopathological characteristics were retrospectively gathered. Testing for RET fusion status was conducted using next-generation sequencing (NGS). The ORR was calculated as the proportion of patients who achieved either a complete response (CR) or partial response (PR) to the treatment. The disease control rate (DCR) was determined by the percentage of patients who had a CR, PR, or stable disease (SD). PFS was defined as the period from the start of the treatment to when progressive disease (PD) was noted or the occurrence of death. OS was measured from the time treatment began until the patient passed away. For those who were still alive at their last appointment, the information was censored.

RET fusions were identified using a polymerase chain reaction (PCR) panel or next-generation sequencing (NGS). The expression levels of PD-L1 were evaluated in tumor tissues using immunohistochemistry (IHC) with the DAKO 22C3 PharmDx antibody. The tumor proportion score (TPS), indicating the percentage of tumor cells showing partial or complete membrane staining positivity, was used to measure PD-L1 levels. Additionally, patients were categorized based on their PD-L1 expression into negative, low, or high groups, corresponding to TPSs of < 1%, 1–49%, and ≥ 50%, respectively. Tumor mutational burden (TMB) was also assessed through NGS, covering a minimum of 300 genes.

### Statistical analysis

Statistical analyses were conducted using SPSS version 21.0 (IBM Corp., USA). Categorical data were analyzed using Fisher’s exact test and the Wilcoxon two-sample test. A P-value of less than 0.05 was considered statistically significant. Survival curves were plotted using the Kaplan-Meier method, and survival differences were assessed using the stratified log-rank test. Hazard ratios (HRs) and 95% CIs were computed using Cox proportional hazards regression analysis.

## Results

### Patients’ clinicopathological features

**Fig. 1:**
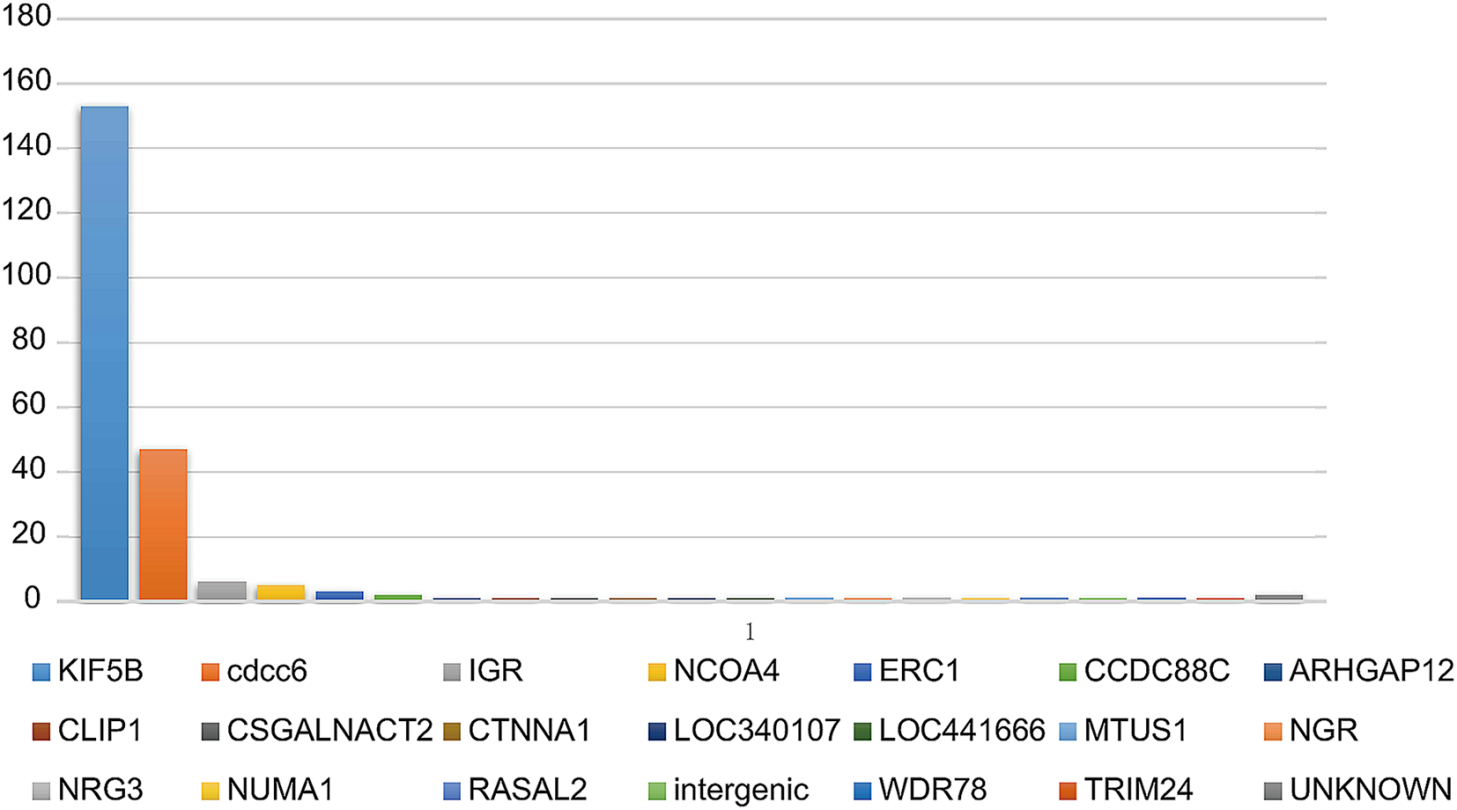
The fusion partners of RET gene

Between April 2017 and January 2022, a total of 232 patients with RET-fusion positive NSCLC were retrospectively included in this study from the First Affiliated Hospital of Zhengzhou University. Among them, 84 were diagnosed with stage I, there were no patients with stage II, 34 with stage III, and 114 with stage IV disease, as shown in Table 1. At the time of their metastatic disease diagnosis, 21 patients (9.1%) had brain metastases. The majority of patients (224 out of 232, 96.7%) were diagnosed with adenocarcinoma; other histological types included neuroendocrine carcinoma (5 patients), squamous cell carcinoma (2 cases), and one case of a sclerosing pneumocytic tumor. A large proportion, specifically 187 patients (81.0%), were never smokers. Moreover, the median age was 59 years, ranging from 30 to 88, aligning with the typical clinical profile of patients with RET-fusion positive NSCLC. KIF5B was identified as the most common fusion partner. Other partners included CCDC6, IGR4, TRIM, among others, which are illustrated in Fig. 1. In this cohort, 27 patients were found to carry both TP53 and RET fusion mutations. Also, 5 patients were found to have concurrent EGFR sensitizing mutations, which included 3 cases with an exon 19 deletion and 2 cases with an exon 21 L858R mutation, as documented in Supplemental Table 1. RET fusion testing was conducted using NGS or PCR in 209 patients (90.1%) and 23 patients (9.9%), respectively. The treatment approaches and medications utilized in the study are outlined in Supplemental Table 2.

**Table 1:** Patients’ characteristics

Characteristics	Total RET-fusion (*n* = 232)	Immunophenotyped cohort (*n* = 194)	Treatment cohort (*n* = 38)	*P*-value
**Median age (range), years**	59(30–88)	59(30–88)	57 (39–71)	0.62
**Sex**				0.87
Male	100 (43.1)	85 (43.8)	15 (39.5)	
Female	132 (56.9)	109 (56.2)	23 (60.5)	
**Smoking history**				0.84
No	188 (81.0)	156 (80.4)	32 (84.2)	
Yes	44 (19.0)	38 (19.6)	6 (15.8)	
**Histology**				0.08
Adenocarcinoma	224 (96.6)	189 (97.4)	35 (92.1)	
Squamous	2 (0.9)	1 (0.5)	1 (2.6)	
Neuroendocrine carcinoma	3 (1,3)	1 (0.5)	2 (5.3)	
Atypical carcinoid	2 (0.9)	2 (1.0)	0 (0)	
Others	1 (0.4)	1 (0.5)	0 (0)	
**Stage**				0.000
I	84 (36.2)	84 (43.3)	0 (0)	
II	0 (0)	0 (0)	0 (0)	
III	34 (14.7)	31 (16.0)	3 (7.9)	
IV	114 (49.1)	79 (40.7)	35 (92.1)	
**Brain metastases**				0.000
Yes	21 (9.1)	8 (4.1)	13 (34.2)	
No	211 (90.9)	186 (95.9)	25 (65.8)	
**ICI combination therapy**				
First line			17 (44.7)	
Second or later line			21 (55.3)	

### Expression of PD-L1 and TMB status in RET + NSCLC patients

PD-L1 expression levels were evaluated in 129 individuals with RET fusion-positive NSCLCs, including 28 participating in the treatment cohort and 101 in the immunophenotypic cohort. The division of PD-L1 expression levels across these patients showed that 65 (50.8%) exhibited negative PD-L1 expression (under 1%), 40 (31.3%) had low levels (between 1 and 49%), and 23 (17.9%) demonstrated high expression (50% or greater), detailed in Table 2. TMB was assessed for 35 patients with RET fusion-positive NSCLCs—8 from the treatment cohort and 27 from the immunophenotypic cohort. Among those assessed, the majority, 30 patients, had a TMB lower than 10 mutations per megabase (mut/mb) (85.7%), while 5 (14.3%) displayed a TMB of 10 mut/mb or higher. To ensure a consistent comparison of TMB levels, analyses were limited to those patients who received both NGS and TMB testing via the MSK-IMPACT panel, which was the most commonly implemented panel. The median TMB observed in lung cancers with RET alterations stood at 5.2 mut/mb, ranging from 0 to 17.5.

**Table 2:** PD-L1 expression and TMB status in RET fusion positive patients

	Total RET-fusion positive	Immunophenotyped cohort	ICI treatment cohort	*P*-value
**PD-L1 expression TPS (%)**	*N* = 129	*N* = 101	*n* = 28	0.07
Negative (< 1)	66 (51.2)	53 (52.5)	13 (25.0)	
low (1–49)	40 (31.0)	32 (31.7)	8 (25.0	
High (≥ 50)	23 (17.8)	16 (15.8)	7 (50.0)	
**PD-L1 expression TPS (%) median**	0	0	30	0.08
TMB (mut/Mb)	*N* = 35	*N* = 27	*n* = 8	0.28
Low (< 5)	22 (62.9)	18 (66.7)	4 (50)	
Intermediate (5–10)	8 (22.9)	4 (14.8)	4 (50)	
High (≥ 10)	5 (14.3)	5 (18.5)	0 (0)	
**TMB (mut/Mb)**				0.005
Median (range)	3.99 (1.0-12.7)	3.1 (1.0-12.7)	5.3 (3.5–7.01)	

### Response to ICIs in RET + NSCLCs

**Fig. 2:**
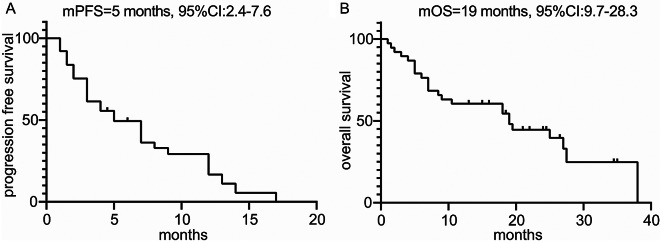
PFS and OS of RET fusion positive NSCLC patients treated with ICI based therapies. (**A**) the mPFS of RET fusion positive NSCLC patients treated with ICIs; (**B**) the mOS of RET fusion positive NSCLC patients treated with ICIs

**Fig. 3:**
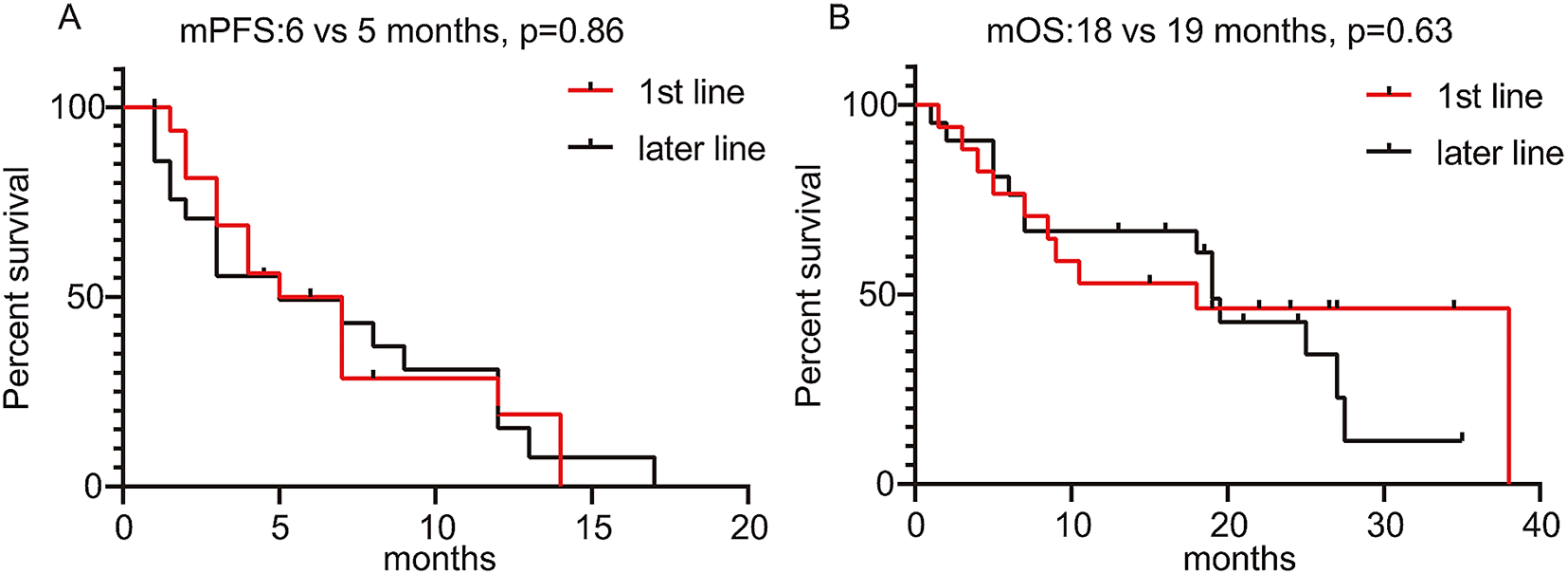
PFS and OS of ICIs stratified by treatment lines. (**A**) PFS for patients of the treatment cohort stratified by treatment line; (**B**) OS for patients of the treatment cohort stratified by treatment line

In this series, a total of 38 patients were treated with ICIs; patients who did not receive ICIs (administered with chemotherapy, antiangiogesis agents or RET TKIs or giving up therapy) were not included in this analysis. Details of the screening workup are summarized in supplemental Fig. 1. Out of these, 17 patients were given ICI combinations as a first-line treatment, while 21 patients received ICIs in later lines of treatment. Among them, 9 patients achieved a PR, though no CR were observed at the time of data cutoff. The ORRwas 23.7% (9 out of 38 patients, with a 95% CI of 9.5-37.8%). The ORR for patients treated with ICIs as a first-line therapy was 41.2% (95% CI, 15.1-67.3%), notably higher compared to the 14.3% (95% CI, 2.0-30.6%) for those treated in the second or later lines of therapy (*p* = 0.022). However, the DCR showed no significant difference between patients treated in the first line versus those in the second or subsequent lines (82.4%, 95% CI, 62.1-93.7% vs. 81.0%, 62.6-99.3%, *p* = 0.64). The median PFS was 5 months (95% CI, 2.4–7.6 months) for all patients receiving ICIs, as seen in Fig. 2A. Additionally, the median OS was 19 months (95% CI, 9.7–28.3 months), depicted in Fig. 2B. When analyzed by the line of treatment, the median PFS for patients treated with ICIs as a first line was slightly longer than for those treated beyond the first line (6 months vs. 5 months, *p* = 0.86, Hazard Ratio [HR] = 0.93, 95% CI, 0.43–2.04), but this difference was not statistically significant, as illustrated in Fig. 3A. Similarly, no statistical significance was found in median OS between patients receiving ICIs as a first line compared to those in the second or later lines (18 months vs. 19 months, *p* = 0.63), as shown in Fig. 3B.

### The correlation between ICI response and PD-L1 levels and TMB status

**Fig. 4:**
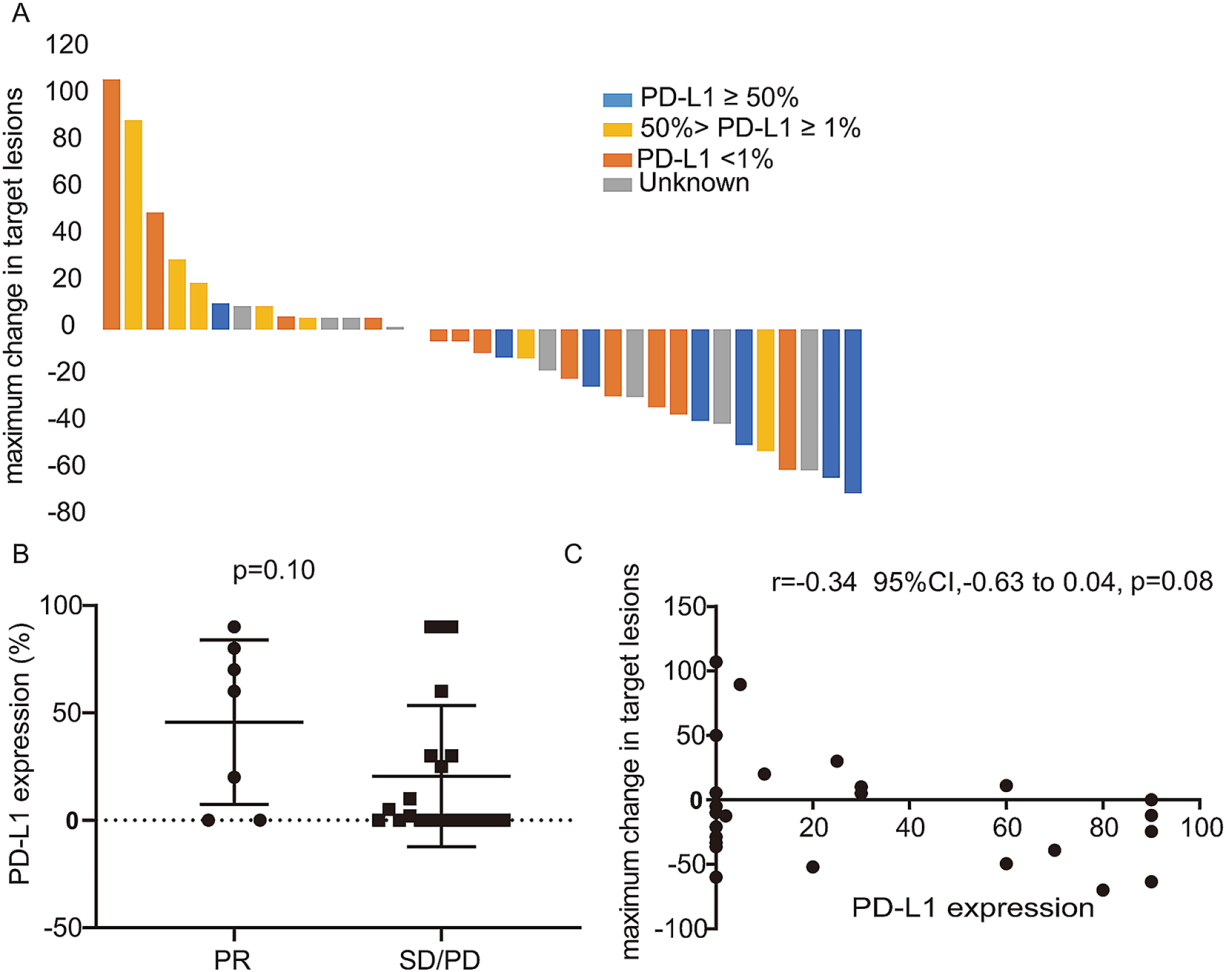
The correlation between activities of ICIs in RET fusion positive patients and PD-L1 level. (**A**) maximum Changes in the target lesions based on different PD-L1 levels (**B**) relationship between PD-L1 expression and efficacy in responders (PR) and non-responders (SD and PD); (**C**) The correlation between PD-L1 level and maximum changes in target lesions

Previous studies have indicated that PD-L1 levels and TMB status may correlate with the effectiveness of ICIs [20, 21]. Consequently, in this study, we investigated the predictive value of PD-L1 expression and TMB status for ICI response in patients with RET fusion-positive NSCLC. A waterfall plot demonstrated the maximum change in target lesions from baseline, categorized by PD-L1 expression levels (Fig. 4A). Upon analyzing the relationship between PD-L1 expression levels and treatment efficacy, we found no significant association between PD-L1 expression levels and response (median 45% versus 20%, *p* = 0.10, Fig. 4B) when comparing responders (those achieving a PR) to non-responders (those with SD or PD). Furthermore, there was no statistical correlation between the maximum change in the sum of target lesions and PD-L1 levels (*r*=-0.34, 95% CI, -0.63-0.04, *p* = 0.08, Fig. 4C). Due to the limited number of cases, we could not assess the correlation between response and TMB. Nevertheless, there was an observed trend towards improved PFS in patients with positive PD-L1 expression (7 months for PD-L1 ≥ 1% versus 3 months for PD-L1 < 1%, *p* = 0.27), as shown in the additional file, Figure S3A. However, this trend did not extend to the correlation between OS and PD-L1 level, as indicated in the additional file, Figure S3B.

### Adverse events profile

Out of the patients receiving ICIs, 75.7% (28 patients) experienced adverse events, as outlined in Supplemental Table 4. However, only a small fraction of these cases, 17.9% (5 out of 28 patients), involved severe toxicities (grade 3 or higher adverse events). Overall, the side effect profile was considered manageable. Adverse events with an occurrence greater than 20% included liver injury (35.7%), anemia (57.1%), leukopenia (21.4%), and skin effects (21.4%), with additional adverse events detailed in Supplemental Table 4. Five patients developed grade 3 or higher adverse effects: one with anemia, one with leukopenia, one with thrombocytopenia, and two with liver injury. There were no cases of Interstitial Lung Disease reported in this series.

## Discussion

To our knowledge, this study represents the most extensive cohort of RET fusion-positive NSCLC patients in China, detailing the immunophenotype and the effectiveness of ICI-based treatments. The research included data from 232 lung cancer patients with RET fusions, gathered from real-world clinical settings. However, due to the study’s limitations and its retrospective nature, there is a clear need for randomized, prospective trials to corroborate our findings. Our cohort exhibited several distinctive characteristics that are worth noting. Firstly, the majority of our patients (187, or 80.3%) were never smokers, meaning they either never had a smoking history or had quit smoking at least 20 years prior. This finding contradicts previous studies, which indicated that 29-50% of patients with RET fusion mutations were smokers, although those studies included only a limited number of cases. Secondly, while most of the patients were diagnosed with adenocarcinoma, a small subset of 3.4% had other histological types. This is in line with existing literature that describes the occurrence of RET fusion in NSCLC [22–24]. Notably, in both this series and prior reports, neuroendocrine carcinomas were the most commonly diagnosed lung cancers with RET fusions, aside from adenocarcinoma [22]. Additionally, over half of our patients were diagnosed with stage IV disease, aligning with findings from previous research [25]. The thorax and bones were the most common sites of metastasis identified at the initial diagnosis. However, only 7 patients (3%) had adrenal metastases at the time of their initial diagnosis, which is lower compared to the higher incidence typically seen in NSCLC. Our study also observed that 9% of patients had brain metastases at the initial diagnosis, which is less than the percentage reported in prior studies [26]. One possible explanation for this discrepancy could be that baseline screening for brain metastases was not standardized.

In our research, the majority of patients with RET fusion-positive mutations showed PD-L1 expression of less than 50% (17.8%). Currently, the reports on PD-L1 expression levels in patients with RET fusion-positive mutations are mixedt [27, 28]. One small-scale study found a higher proportion of RET mutation-positive patients to have elevated levels of PD-L1 expression [27], On the other hand, another study came to an opposite conclusion, suggesting that only a minority of patients exhibited high PD-L1 expression [28].

In our series, we observed that the majority of patients with RET-fusion mutations had a TMBof less than 10 mutations per megabase. The median TMB in our RET-altered patients was significantly lower than that seen in other types of lung cancers, including those driven by other oncogenic mutations, which are typically found in non-smokers [27, 29]. Prior research has indicated that patients with a history of smoking tend to have a higher TMB [30, 31], which may partly explain the lower TMB observed in patients with RET alterations. Moreover, these findings are consistent with those reported in the literature. Given this immunophenotype, it is not surprising that ICI monotherapy would have a lower response rate and a shorter period of PFS, as documented in previous research. Nevertheless, our data suggest that combining ICIs with chemotherapy also resulted in suboptimal effectiveness (ORR: objective response rate of 27.0%) and limited PFS (median PFS of 5 months). These results reinforce the notion that patients with RET-fusion positive tumors may not derive benefits from ICIs, neither as a standalone treatment nor in combination with chemotherapy.

Prior research has indicated that patients with oncogenic driver mutations, such as EGFR sensitizing mutations and ALK fusions, generally do not benefit greatly from ICI monotherapy, leading clinical trials on ICIs to typically exclude these groups [12, 27, 32]. In line with this, our data also demonstrated that patients with RET fusions had a limited response to treatment combinations that included ICIs [12, 33, 34]. This aligns with the results from other small-scale studies. Yet, contrasting findings have emerged from a recent study suggesting that RET mutations might actually offer a positive predictive response to ICI therapy [35], proposing that RET translocations and RET point mutations may trigger distinct molecular pathways in cancer. Importantly, previous research has suggested that NSCLCs positive for RET fusions are more likely to respond to platinum-based chemotherapy, particularly when combined with pemetrexed [36]. This study discovered that among 18 NSCLC patients with RET fusions treated with pemetrexed, there was a 45% objective response rate (ORR) and a median PFS of 19 months [36], These outcomes are on par with similar treatment regimens used for ROS1 fusion-positive and ALK-rearranged NSCLCs. Additionally, further findings have shown that pemetrexed-based regimens also yield favorable results in RET fusion-positive NSCLCs, albeit to a lesser extent, with a median PFS of 9 months reported in another study [24]. Our results indicated that combining ICIs with chemotherapy provided limited benefit in RET fusion-positive NSCLCs. Therefore, considering all these factors together, it appears that ICIs may affect the effectiveness of chemotherapy. More research is needed to verify these findings.

Certainly, this research has several limitations that should be highlighted for clarity. The primary concern is the small sample size and the retrospective nature of the study. Secondly, the lack of centralized confirmation of RET fusion status and the diversity in molecular testing methods are noteworthy issues. It’s important to recognize that most patients were tested for RET fusions at initial diagnosis, while some were tested upon relapse. However, due to the retrospective nature of the study, we can’t precisely determine the timing and reasons why some individuals did not undergo RET testing at initial diagnosis, especially as some were diagnosed with stage IV disease. Additionally, there was no systematic requirement for PD-L1 testing and TMB status evaluation in patients with RET fusion-positive tumors. Furthermore, the study did not employ a central independent imaging review to determine tumor response, which was instead assessed by the investigators. Therefore, the conclusions reached by this study should be approached with caution.

In conclusion, our study indicates that patients with NSCLC harboring RET fusions are typically non-smokers diagnosed with adenocarcinoma. The immunophenotype of RET-positive NSCLCs is characterized by limited PD-L1 expression and a low to intermediate TMB status, suggesting they are “cold” tumors less likely to respond to immunotherapy. Our data also suggest that combining ICIs with chemotherapy does not improve efficacy compared to ICI monotherapy. Moreover, the presence of ICIs might even negatively influence the effectiveness of chemotherapy. These results reinforce the notion that patients with RET fusion-positive NSCLC may not derive benefit from ICI treatments and should potentially be excluded from such therapies. However, further studies are needed to substantiate these findings.

### Electronic supplementary material

Below is the link to the electronic supplementary material.

Supplementary Material 1

## Data Availability

The datasets used and/or analyzed during the current study are available from the corresponding author upon reasonable request.

## References

[1] Sholl LM, Aisner DL, Varella-Garcia M, Berry LD, Dias-Santagata D, Wistuba II (2015). Multi-institutional Oncogenic Driver Mutation Analysis in Lung Adenocarcinoma: the Lung Cancer Mutation Consortium Experience. J Thorac Oncol.

[2] Steuer CE, Behera M, Berry L, Kim S, Rossi M, Sica G (2016). Role of race in oncogenic driver prevalence and outcomes in lung adenocarcinoma: results from the Lung Cancer Mutation Consortium. Cancer.

[3] Adashek JJ, Desai AP, Andreev-Drakhlin AY, Roszik J, Cote GJ, Subbiah V (2021). Hallmarks of RET and co-occuring genomic alterations in RET-aberrant cancers. Mol Cancer Ther.

[4] Ferrara R, Auger N, Auclin E, Besse B (2018). Clinical and translational implications of RET rearrangements in Non-small Cell Lung Cancer. J Thorac Oncol.

[5] Osta BE, Ramalingam SS (2020). RET Fusion: joining the ranks of Targetable Molecular drivers in NSCLC. JTO Clin Res Rep.

[6] Drilon A, Wang L, Hasanovic A, Suehara Y, Lipson D, Stephens P (2013). Response to Cabozantinib in patients with RET fusion-positive lung adenocarcinomas. Cancer Discov.

[7] Cascetta P, Sforza V, Manzo A, Carillio G, Palumbo G, Esposito G et al. RET Inhibitors in Non-Small-Cell Lung Cancer. *Cancers (Basel)* 2021, 13(17). 10.3390/cancers13174415 PMC8431193 34503226

[8] Lee SH, Lee JK, Ahn MJ, Kim DW, Sun JM, Keam B (2017). Vandetanib in pretreated patients with advanced non-small cell lung cancer-harboring RET rearrangement: a phase II clinical trial. Ann Oncol.

[9] Gainor JF, Curigliano G, Kim DW, Lee DH, Besse B, Baik CS (2021). Pralsetinib for RET fusion-positive non-small-cell lung cancer (ARROW): a multi-cohort, open-label, phase 1/2 study. Lancet Oncol.

[10] Drilon A, Oxnard GR, Tan DSW, Loong HHF, Johnson M, Gainor J (2020). Efficacy of Selpercatinib in RET Fusion-positive non-small-cell Lung Cancer. N Engl J Med.

[11] Zhou C, Solomon B, Loong HH, Park K, Pérol M, Arriola E (2023). First-line selpercatinib or chemotherapy and Pembrolizumab in RET Fusion-positive NSCLC. N Engl J Med.

[12] Mazieres J, Drilon A, Lusque A, Mhanna L, Cortot AB, Mezquita L (2019). Immune checkpoint inhibitors for patients with advanced lung cancer and oncogenic driver alterations: results from the IMMUNOTARGET registry. Ann Oncol.

[13] Lisberg A, Cummings A, Goldman JW, Bornazyan K, Reese N, Wang T (2018). A phase II study of Pembrolizumab in EGFR-Mutant, PD-L1+, tyrosine kinase inhibitor Naïve patients with Advanced NSCLC. J Thorac Oncol.

[14] Seegobin K, Majeed U, Wiest N, Manochakian R, Lou Y, Zhao Y (2021). Immunotherapy in Non-small Cell Lung Cancer with actionable mutations other Than EGFR. Front Oncol.

[15] Dudnik E, Bshara E, Grubstein A, Fridel L, Shochat T, Roisman LC (2018). Rare targetable drivers (RTDs) in non-small cell lung cancer (NSCLC): outcomes with immune check-point inhibitors (ICPi). Lung Cancer.

[16] Guisier F, Dubos-Arvis C, Viñas F, Doubre H, Ricordel C, Ropert S (2020). Efficacy and safety of Anti-PD-1 immunotherapy in patients with Advanced NSCLC with BRAF, HER2, or MET mutations or RET translocation: GFPC 01-2018. J Thorac Oncol.

[17] Baglivo S, Ludovini V, Moretti R, Bellezza G, Sidoni A, Roila F (2020). RET rearrangement as a predictor of unresponsiveness to Immunotherapy in Non-small Cell Lung Cancer: report of two cases with review of the literature. Oncol Ther.

[18] Hegde A, Andreev-Drakhlin AY, Roszik J, Huang L, Liu S, Hess K (2020). Responsiveness to immune checkpoint inhibitors versus other systemic therapies in RET-aberrant malignancies. ESMO Open.

[19] Offin M, Guo R, Wu SL, Sabari J, Land JD, Ni A et al. Immunophenotype and response to Immunotherapy of RET-Rearranged lung cancers. JCO Precis Oncol 2019, 3. 10.1200/PO.18.00386 PMC6561651 31192313

[20] Reck M, Rodríguez-Abreu D, Robinson AG, Hui R, Csőszi T, Fülöp A (2016). Pembrolizumab versus Chemotherapy for PD-L1-Positive non-small-cell Lung Cancer. N Engl J Med.

[21] Marabelle A, Fakih M, Lopez J, Shah M, Shapira-Frommer R, Nakagawa K (2020). Association of tumour mutational burden with outcomes in patients with advanced solid tumours treated with pembrolizumab: prospective biomarker analysis of the multicohort, open-label, phase 2 KEYNOTE-158 study. Lancet Oncol.

[22] Aldea M, Marinello A, Duruisseaux M, Zrafi W, Conci N, Massa G (2023). RET-MAP: an International Multicenter Study on Clinicobiologic Features and treatment response in patients with Lung Cancer harboring a RET Fusion. J Thorac Oncol.

[23] Ju YS, Lee WC, Shin JY, Lee S, Bleazard T, Won JK, et al. A transforming KIF5B and RET gene fusion in lung adenocarcinoma revealed from whole-genome and transcriptome sequencing. Genome Res. 2012;22(3):436–45. 10.1101/gr.133645.111 PMC3290779 22194472

[24] Lee J, Ku BM, Shim JH, La Choi Y, Sun JM, Lee SH (2020). Characteristics and outcomes of RET-rearranged Korean non-small cell lung cancer patients in real-world practice. Jpn J Clin Oncol.

[25] Wang R, Hu H, Pan Y, Li Y, Ye T, Li C (2012). RET fusions define a unique molecular and clinicopathologic subtype of non-small-cell lung cancer. J Clin Oncol.

[26] Drilon A, Lin JJ, Filleron T, Ni A, Milia J, Bergagnini I (2018). Frequency of brain metastases and multikinase inhibitor outcomes in patients with RET-Rearranged lung cancers. J Thorac Oncol.

[27] Negrao MV, Skoulidis F, Montesion M, Schulze K, Bara I, Shen V et al. Oncogene-specific differences in tumor mutational burden, PD-L1 expression, and outcomes from immunotherapy in non-small cell lung cancer. J Immunother Cancer 2021, 9(8). 10.1136/jitc-2021-002891 PMC8356172 34376553

[28] Lu C, Dong XR, Zhao J, Zhang XC, Chen HJ, Zhou Q (2020). Association of genetic and immuno-characteristics with clinical outcomes in patients with RET-rearranged non-small cell lung cancer: a retrospective multicenter study. J Hematol Oncol.

[29] Jiang B, Hu L, Dong D, Guo Z, Wei W, Wang C (2023). TP53 or CDKN2A/B covariation in ALK/RET/ROS1-rearranged NSCLC is associated with a high TMB, tumor immunosuppressive microenvironment and poor prognosis. J Cancer Res Clin Oncol.

[30] Nagahashi M, Sato S, Yuza K, Shimada Y, Ichikawa H, Watanabe S (2018). Common driver mutations and smoking history affect tumor mutation burden in lung adenocarcinoma. J Surg Res.

[31] Wang X, Ricciuti B, Nguyen T, Li X, Rabin MS, Awad MM (2021). Association between Smoking History and Tumor Mutation Burden in Advanced Non-small Cell Lung Cancer. Cancer Res.

[32] Madeddu C, Donisi C, Liscia N, Lai E, Scartozzi M, Macciò A. EGFR-Mutated Non-small Cell Lung Cancer and Resistance to Immunotherapy: role of the Tumor Microenvironment. Int J Mol Sci 2022, 23(12). 10.3390/ijms23126489 PMC9224267 35742933

[33] Bhandari NR, Hess LM, Han Y, Zhu YE, Sireci AN (2021). Efficacy of immune checkpoint inhibitor therapy in patients with RET fusion-positive non-small-cell lung cancer. Immunotherapy.

[34] Akers KG, Oskar S, Zhao B, Frederickson AM, Arunachalam A. Clinical outcomes of PD-1/PD-L1 inhibitors among patients with Advanced or Metastatic Non-small Cell Lung Cancer with BRAF, ERBB2/HER2, MET, or RET alterations: a systematic literature review. J Immunother 2023. 10.1097/CJI.0000000000000500 PMC10984634 38112201

[35] Long JY, Li RZ, Wang DX, Liu H, Tian J, Ding ZN (2023). Comprehensive molecular analysis identifies RET alterations association with response of ICIs in multi-immunotherapy cohorts. Int Immunopharmacol.

[36] Drilon A, Bergagnini I, Delasos L, Sabari J, Woo KM, Plodkowski A (2016). Clinical outcomes with pemetrexed-based systemic therapies in RET-rearranged lung cancers. Ann Oncol.

