# Supplementary material for: Response to immune checkpoint inhibitor combination therapy in metastatic RET-mutated lung cancer from real-world retrospective data
Source: BMC Cancer. 2024 Feb 5;24:178. doi: 10.1186/s12885-024-11852-3 (PMC10845679; doi:10.1186/s12885-024-11852-3)
Supplement: Supplementary file 1 — Supplementary Material 1 [file 12885_2024_11852_MOESM1_ESM.docx]

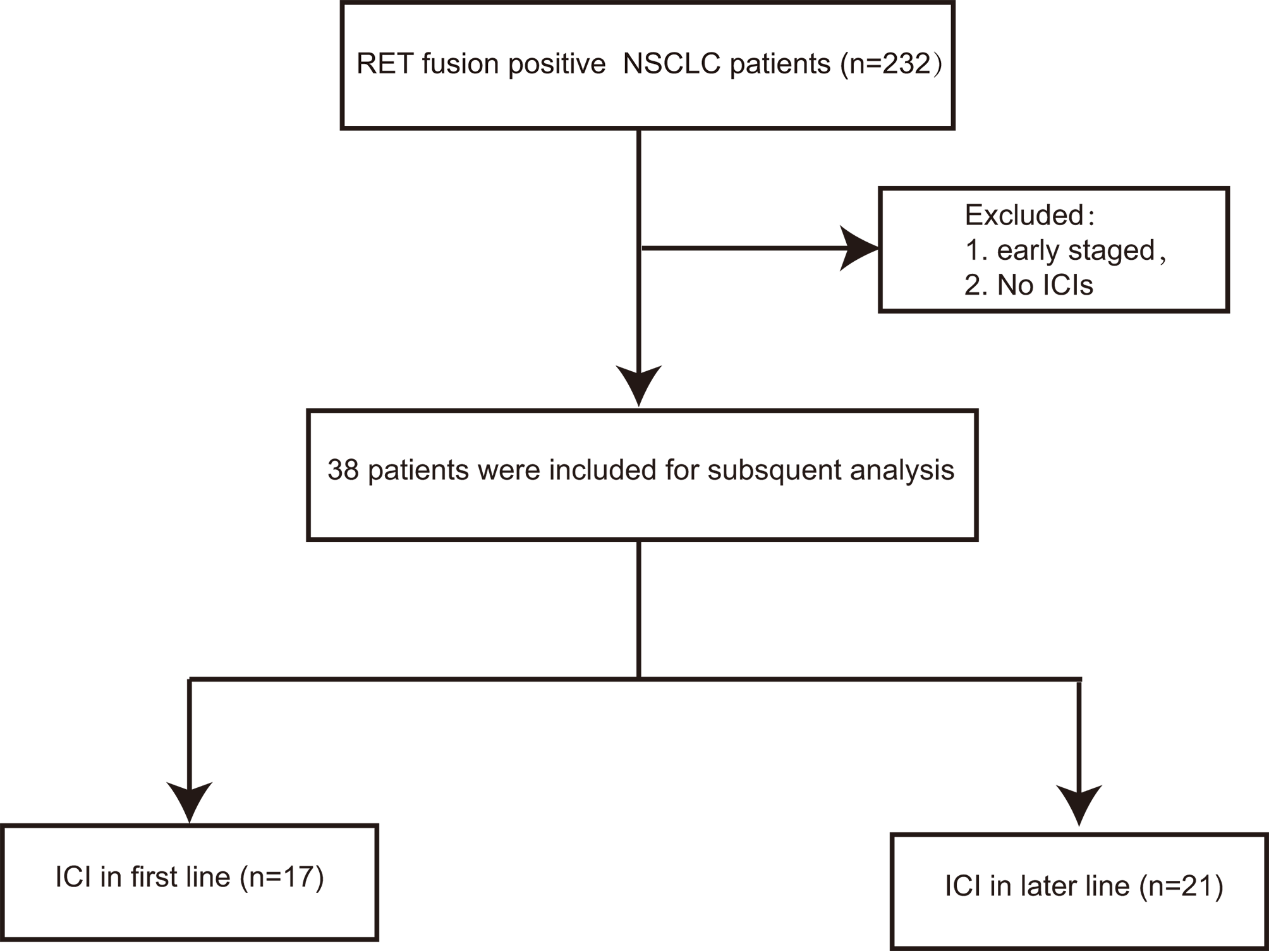


Supplemental figure 1 flow graph of the study


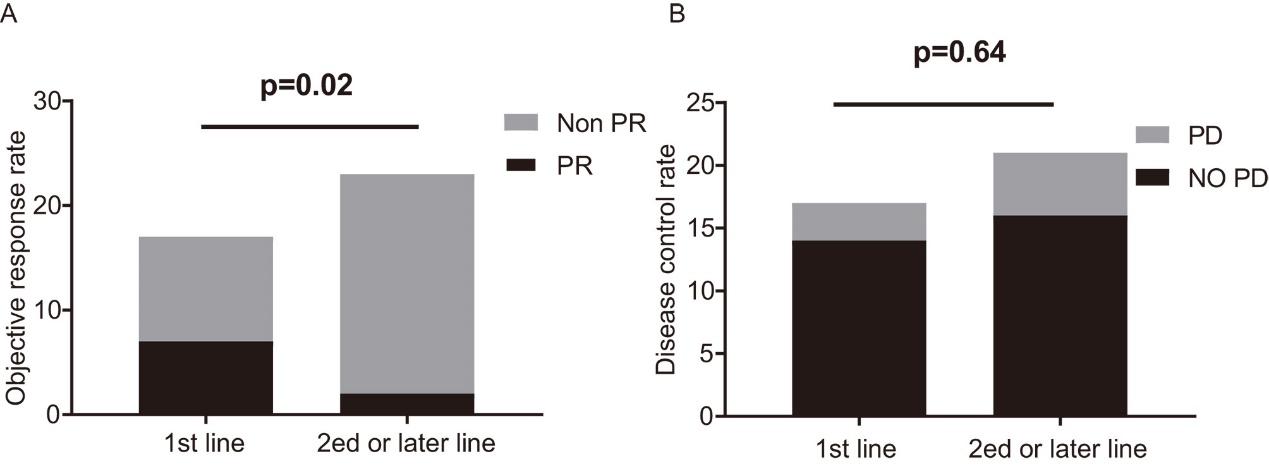


Supplemental figure 2 Response of ICI-treated RET-positive patients stratified by treatment lines. A and B: ORR and DCR for patients in different treatment lines. ORR, objective response rate, including partial response and complete response; DCR, disease control rate, including stable disease, partial response, and complete response.


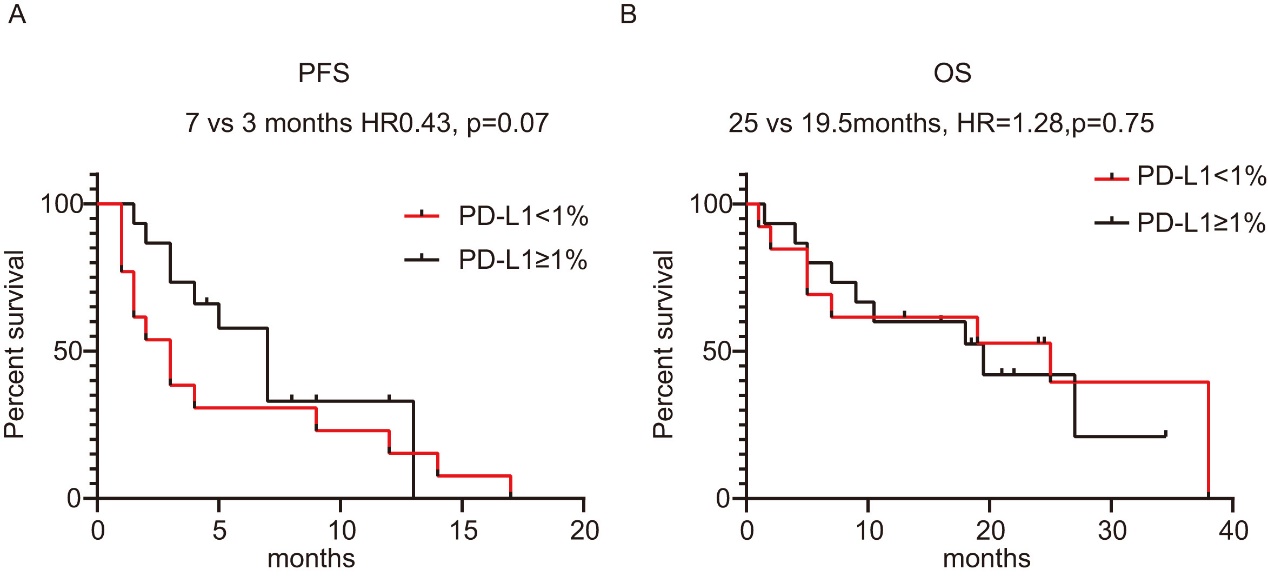


Supplemental figure 3 PFS and OS for patients stratified by PD-L1 expression. A and B: PFS and OS of ICI-treated patients based on PD-L1 expression. PFS, progression-free survival; OS, overall survival.

Supplemental table 1 Co-occurring mutations with RET fusions

| Co-occurring mutations | Total population |
| --- | --- |
| TP53 | 27/232 (11.6%) |
| KRAS G12D | 2/232 (0.8%) |
| NRAS | 2/232 (0.8%) |
| MTOR | 6/232 (2.6%) |
| HRAS | 2/232 (0.8%) |
| ERBB2 | 4/232 (1.7%) |
| PTEN | 7/232 (3.0%) |
| CDKN2A | 7/232 (3.0%) |
| EGFR  Exon 19 deletion  Exon 21 L858R | 3/232 (1.2%)  2/232 (0.8%) |
| ARID1A | 4/232 (1.7%) |
| RET mutations | 4/232 (1.7%) |

Supplemental Table 2: Treatment regimens for patients treated with ICIs.

| ID | Regimen | Line | PFS (mo) | Reason for discontinuation | Best response |
| --- | --- | --- | --- | --- | --- |
| 1 | Tislelizumab + chemo | 1 | 3 | PD | SD |
| 2 | Camrelizumab + chemo | 1 | 3 | PD | PR |
| 3 | Chemo + Camrelizumab | 1 | 12 | Ongoing | PR |
| 4 | sintilimab+ chemo | 1 | 8 | ongoing | SD |
| 5 | Camrelizumab + chemo | 1 | 14 | PD | PR |
| 6 | Tislelizumab + chemo | 2 | 9 | ongoing | SD |
| 7 | toripalimab + chemo | 1 | 2 | PD | PD |
| 8 | Camrelizumab+chemo | 2 | 4.5 | ongoing | SD |
| 9 | Camrelizumab +chemo | 1 | 1.5 | PD | PD |
| 10 | pembrolizumab + chemo | 1 | 4 | PD | PR |
| 11 | Camrelizumab + chemo | 2 | 1 | PD | PD |
| 12 | Camrelizumab +chemo | 1 | 2 | PD | PD |
| 13 | Camrelizumab +chemo | 2 | 3 | ongoing | SD |
| 14 | Camrelizumab + chemo | 1 | 4 | PD | SD |
| 15 | Camrelizumab + chemo | 2 | 5 | PD | SD |
| 16 | Camrelizumab + chemo | 1 | 1 | PD | PD |
| 17 | Tislelizumab +chemo | 1 | 6 | ongoing | SD |
| 18 | Camrelizumab + chemo | 2 | 2 | PD | PD |
| 19 | Camrelizumab + chemo | 1 | 5 | PD | PR |
| 20 | sintilimab + chemo | 3 | 7 | PD | SD |
| 21 | Camrelizumab + chemo | 1 | 12 | PD | SD |
| 22 | Camrelizumab + chemo | 2 | 9 | PD | SD |
| 23 | Camrelizumab + chemo | 2 | 12 | PD | SD |
| 24 | Sintilimab + chemo | 1 | 7 | PD | PR |
| 25 | Camrelizumab + chemo | 1 | 7 | PD | SD |
| 26 | Camrelizumab + chemo | 3 | 17 | PD | SD |
| 27 | Camrelizumab + chemo | 3 | 3 | PD | SD |
| 28 | Camrelizumab + chemo | 2 | 3 | PD | SD |
| 29 | Camrelizumab + chemo | 2 | 1 | PD | PD |
| 30 | Camrelizumab + chemo | 2 | 12 | PD | PR |
| 31 | Camrelizumab+chemo | 4 | 1 | PD | PD |
| 32 | Camrelizumab + chemo | 3 | 1 | SD | SD |
| 33 | Camrelizumab + chemo | 3 | 1.5 | PD | PD |
| 34 | Camrelizumab+chemo | 2 | 3 | PD | SD |
| 35 | Camrelizumab + chemo | 1 | 7 | PD | PR |
| 36 | Camrelizumab+chemo | 2 | 8 | PD | PD |
| 37 | sintilimab + chemo | 3 | 1.5 | PD | PD |
| 38 | sintilimab + chemo | 3 | 13 | PD | PR |

chemo, chemotherapy; PFS, progression free survival; mo, months; PD, progressive disease; PR, partial response; SD, stable disease; NA, not available.

Supplemental Table 3 the efficacy of ICI based therapies in patients with RET fusions

| Best response | All patients n% | ICI in 1^st^ line | ICI in 2ed or later line |
| --- | --- | --- | --- |
| CR | 0 | 0 | 0 |
| PR | 9 (23.7%) | 7 (41.2%) | 2 (9.5%) |
| SD | 21 (55.3%) | 7 (41.2%) | 14 (66.7%) |
| PD | 8 (21.1%) | 3 (17.6%) | 5 (23.8%) |
| ORR | 9 (23.7%; 95%CI, 9.5%-37.8%) | 7 (41.2%; 95% CI: -15.1%-67.3%) | 2 (9.5%;95% CI: 4.2%-23.2%) |
| DCR | 30 (78.9%, 95%CI: 64.5%-92.5%) | 14 (82.4%;95% CI -62.1%-102.6%) | 16 (76.2%; 95% CI 56.3%-96.1%) |

Supplemental table 4 safety profile

| event | treated population (28 patients have occured side effects) | |
| --- | --- | --- |
|  | Any grade | Grade 3, 4, 5 |
| total | 75.7% | 10.8% |
| Skin effects | 6/28 (21.4%) | 0 |
| RCCEP | 1/28 (3.6%) | 0 |
| anemia | 16/28 (57.1%) | 1/28 (3.6%) |
| neutropenia | 3/28 (10.7%) | 0 |
| thrombocytopenia | 5/28 (17.9%) | 1/28 (3.6%) |
| leukopenia | 6/28 (21.4%) | 1/28 (3.6%) |
| liver injury | 10/28 (35.7%) | 2/28 (7.1%) |
| hypothyroidism | 5/28 (17.9%) | 0 |
| Kidney injury | 2/28 (7.1%) | 0 |
| adrenal insufficiency | 1/28 (3.6%) | 0 |
| hypokalemia | 2/28 (7.1%) | 0 |
| hyperglycemia | 1/28 (3.6%) | 0 |

RCCEP: Reactive cutaneous capillary endothelial proliferation
